# Hijacking the epigenetic mechanisms of *A. baumannii*

**DOI:** 10.22099/MBRC.2024.48801.1899

**Published:** 2024

**Authors:** S. A. Smiline Girija

**Affiliations:** Department of Microbiology, Saveetha Dental College and Hospitals, Saveetha Institute of Medical and Technical Sciences (SIMATS), Saveetha University, Chennai-600 077, Tamilnadu, India

**Keywords:** Epigenetics, Methylation, Acetylation, A. baumannii, Virulence, Epidrugs

## Abstract

Epigenetic mechanisms attribute to the resistance and virulence of *Acinetobacter baumannii* sparking a renewed area of research. Unveiling the targets pertained to the epigenetic modulation in the bacterium would aid in the curbing its complications in various recalcitrant infections. This review thus throws light on the various epigenetic mechanisms exhibited by *A. bauamnnii*, urging the need to implement epigenetic based novel strategies in precision medicine.

## Epigenetic mechanisms in A. baumannii

Aberrant epigenetic mechanisms are known to induce antibiotic resistance in many drug resistant bacteria. In this line, an interesting fact about *Acinetobacter baumannii* is that, it stealthily hijacks the epigenome taking a twist in the host-parasite interactions for its survival in host tissues [1]. In recent years, curbing the menace of the pan-drug resistant *A. baumannii* is a herculean task for both the clinicians and microbiologists in the health-care setting. Fascinating findings on the epigenetic modulations exhibited by *A. baumannii* for its survival in the host tissues, unveil the epigenomic targets and further on the application of the epidrugs. Epigenetic modulations usually involves methylation of DNA, post translational modifications, acetylation/deacetylations and methylation/demethylation of histones. These epigenetic modulations are fortunately reversible and thus the implementation of epidrugs, to treat the recalcitrant *A. baumannii* infections would be a novel approach in precision medicine. 

## Epigenetic modulations in A. baumannii pathogenesis

Only fewer studies have experimentally evidenced the epigenomic control of *A. baumannii* on the host tissues spurring renewed interest on the application of the epidrugs against the same. A recent study on *A. baumannii* SK17 had revealed a highly conserved modification in histone like protein that had caused acetylation of lysine at position 13 playing a vital role in the bacterial transcription through epigenetic alteration. This acetylation alters the stability and the DNA binding kinetics of the histones that may lead to post-translational modifications giving rise to resistant epigenetic traits in *A. baumannii *[2]. Another interesting epigenetic modulation occurs while *A. baumannii* enters in to the host cells for its survival. Being highly invasive it face challenges during its entry into the epithelium, where it has to destroy the cell adhesion molecule junctions involving E-cadherin. In these entry junctions, nuclear expression of the *A. baumannii* transposase (Tnp), causes methylation of the cpG islands on the promoter sites of E-cadherin, leading to the down regulation of its expression, substantiating the bacterial epigenetic mechanism modulating the host’s sophisticated pathways favoring its entry and further establishment of the disease [3].

## Methylation of cpG islands in A. baumannii virulence

Epigenetic alterations also plays a vital role in vesicular trafficking during its infection process in the host cells. *In-vitro* studies on the post-infection survival of *A. baumannii* in HeLa cells after 4hrs of infection have shown the expression of differentially expressed genes (DEGs) which specifically alters the host cell for its successful colonization. This is achieved via the modulation of the host vesicular traffic mediated by various cytoskeleton proteins and transcription factors, involving the epigenetic methylation of the cpG islands in the host cells [4]. Additionally, *Acinetobacter *adenine methyl transferease (*AamA*) impose an epigenetic control in *A. baumannii* through an adenine methylation at a GAATTC site as evidenced in a recombinant strain ATCC 17978 and the protein expressed via this alteration is characterized as a monomer with a two-domain shape and is considered as the best target for the epidrugs [5]. 

## Epigenetic mechanisms involving LncRNAs

In the same line, long non-coding RNA’s (LncRNAs) in *A. baumannii* are involved in many pathogenic and virulent mechanisms and these LncRNAs are known to regulate the expression of various genes involving the epigenomic mechanisms [6]. And in a recent study, LncRNAs of *A. baumanni* was known to regulate the autophagy mechanism by up regulating the growth arrest specific transcript-5 (GAS5), leading to the degradation of STX17, a key molecule in the formation of autophagosomes in both the infected and the cancerous cells.* A. baumannii* thus evades from autophagy, promoting the IFN-α in the organs and tumour tissues alleviating the injury process [7].

## Need for epidrugs

It is thus evident that the host chromatin and various epigenetic mechanisms are exhibited by *A. baumannii*, both for its survival and pathogenesis. The effector proteins expressed out of these epigenetic alterations, though short lived are known to induce a long term epigenetic effect in the host-parasite interactions and may be thus considered as essential targets to curb *A. baumannii* infections. Thus we may propose the application of epidrugs to revert these aberrant epigenetic machinery that could be a flourishing avenue in *A. baumannii* related therapeutic strategies. Basket trials involving the already known epidrugs such as DNMTi, HDMI, BETi, HMTi and HDACi may be designed and implemented among the hospitalized patients in multiple centers. The results of the epigenetic reversion may be further integrated in a common computational platform for further analysis. Evidence based experimental studies related to the epidrugs against *A. baumannii* may be conducted in all the health care settings and research institutes in order to curb the menace of *A. baumannii* infections and further complications in hospitalized patients.

## Conflict of Interest:

None to declare.
